# Modern Sociology

**Published:** 1901-04-27

**Authors:** 


### Modern Sociology.

### ANIMALS ON THE STAGE—AND OFF.

From whatever point of view the question of performing beasts may be considered, there is one aspect of it that merits the consideration of all humane people, viz., the method of training for the stage. And this, if one may credit the statements of Mr. S. L. Bensusan in the *English Illustrated Magazine* (and afterwards in pamphlet form), is one of needless and revolting cruelty.

Mr. Bensusan claims to have collected " from some of our best-known managers certain facts relating to particular troupes of performing dogs. . . . I withhold," he adds, " names and full particulars, but these can easily be given. . . . Many an animal goes through its performance in a state bordering on the insane, with such an obvious terror of doing the wrong thing that it is really surprising how an intelligent audience can avoid seeing the true state of things. . . . The weekly rehearsals are held in wretched, ill-ventilated underground cellars, with an accompaniment of suffering that would shock a slaughterman."

The demand for trained animals has led to the establishment of houses for the purpose, on the Continent; in Southern France, Spain, Portugal, and Italy, sights that would make an Englishman ill pass unnoticed. Intending purchasers of monkeys are warned not to be too cruel, for monkeys are delicate, and retaliate by quickly dying; horses sulk; and stories of the revenge of elephants are familiar to everyone. But bears and dogs are more hardy, and can longer survive ill-treatment. " English law," said a trainer, " does not come between a man and his bears "; and this is unhappily true.

Take two instances of the trainer's methods:—" I went to his rooms on a matter of business," said Mr. X., " and though sentiment isn't in my line, I felt perfectly ill. The practice-room was hung with spiked collars, whips, and other things, of all sizes and shapes. He had lost two of his dogs, and had sent for two more from the Dogs' Home, and was trying to teach one when I went in. He had it in a spiked collar, and the poor beast was too frightened and too stupid to do what he wanted. He was in a rare rage, he lashed, and dragged, and jerked, the dog got more and more terrified, until at last his temper got the complete mastery over him, and he strangled the poor beast! I was quite glad to see him put it out of its misery. He used to come to the performance, and when a new dog missed its tricks once or twice we never saw the dog again; he would say he had lost it, and grin. I think he trains most of his own dogs, and he kills quite a number." Again: " Herr X. is an Austrian, with nearly a dozen performing hounds. On the night of his first performance in London they were brought upon the stage by attendants, closely muzzled. Before the curtain rose Herr X. went to each dog and half-stunned it with a loaded stick. He then gave orders for the muzzles to be taken off and went through his turn, lashing the dazed animals freely with a long whip of hide and wire. Immediately after the curtain fell the animals were muzzled and led away. The stage-manager called Herr X. to him and said that such treatment was infamous, and must not be repeated. The Austrian went into a violent passion and said that he only did in London what he did abroad, and that it was no concern of anybody's. Finding that he would not be allowed to persist in spite of his bluster, he went through future performances with the muzzles on his dogs."

Sometimes the light of day is unexpectedly let in upon a piece of barbarity, and a just and condign punishment may follow, as in the case of the trainer who narrowly escaped lynching at the hands of three or four English workmen; and the fact that this took place at a theatre of varieties where the management is of the very best, proves how little restriction can be exercised even by the best intentioned managers, who can only forbid any sort of cruelty on the stage. Some simple and well-meaning people have been at the pains to ask the trainers whether there is any truth in these allegations of cruelty; they have, of course, been told that kindness is the only form of treatment necessary, and that, in fact, the performing animal's life is one long holiday. "I lof mein animals," said a particularly brutal foreigner, " unt mein vife, she lofs the animals alzo." His wife is, if possible, the more vicious of the two, and they have been known to kill animals during training.

Obviously, further inquiry is needed, and obviously, also, the agency best fitted to pursue such inquiry is the Royal Society for the Prevention of Cruelty to Animals. By its own showing, the Society prosecutes where evidence of cruelty is complete, and Mr. Bensusan states that the secretary, Mr. John Colam, was provided, some time ago, with a list of some of the worst offenders, but that most of these men have visited London since this was done, and have pursued their vile trade with impunity.

About 130 inspectors are employed by the Society, and one fails to see why some systematic course of investigation cannot be carried out by them. It is prepared, we are told, to proceed against trainers " upon receipt of specific information relating to a definite act of cruelty." Then why, in the name of " this most righteous cause of humanity," to quote the Society's own words, do not the inspectors proceed at once to collect such specific information? The Home Office does not depend on chance letters from correspondents, before sending its inspectors to search out infringements of the Factory Acts; and a charity so largely supported by the public, and whose expenditure is over £33,000 yearly, should justify its claim on public support by taking up the cause of this class of animals. " This charity," it says of itself, " stands alone—the defender of defenceless dumb animals." Last year (1899) there were 7,900 convictions; how many, one wonders, were against animal-trainers?

There is no doubt that any movement to secure drastic reform in this matter would receive the warm support of the managers of the best theatres where performing animals are exhibited. " Speaking personally," says Mr. Charles Morton, manager of the Palace Theatre of Varieties in Shaftesbury Avenue, "I should like all performing animal shows to be done away with. Fifty years' experience has taught me that they are attended with cruelty in varying degrees. I will not go into the question of training by kindness; it is possible in theory, and seldom or never apparent in practice. I should welcome any steps that would improve the present condition of things, and would give any assistance I could."

A movement is on foot at present to secure for animals which fall in the public streets the same ambulance advantages as those accorded to human beings; and one of the daily papers states that a clergyman has gone to St. Petersburg to solicit the sympathy and co-operation of the Czar. Here is a more crying injustice; but it is perpetrated in dark places, and a large section of the public derives a transient amusement from its results. Regularly, as the holiday season comes round, people go to laugh at the wonderful feats of elephants, bears, and monkeys, dogs and cats, and even little dainty birds. And they take the children, who, if they knew the other side, if they guessed at the loaded whip, the spiked collar, the kicks, starvation, mutilation, and death, would be the first to cry out in horror.

